# Examining the paradox: increased malaria risk in children under 5 in female-headed households in Nigeria

**DOI:** 10.1186/s12936-024-04997-w

**Published:** 2024-05-31

**Authors:** Si-Yu Xing, Hai-Ting Zhang, Lin-Min Wang, Hong-Zheng Lu, Zhe-Yu Peng, Miao Liu, Chun-Xiao Li, Sheng-Qun Deng

**Affiliations:** 1 Department of Pathogen Biology, Anhui Province Key Laboratory of Zoonoses, The Provincial Key Laboratory of Zoonoses of High Institutions in Anhui, School of Basic Medical Sciences, Anhui Medical University, Hefei, 230032 China; 2 State Key Laboratory of Pathogen and Biosecurity, Beijing Institute of Microbiology and Epidemiology, Beijing, 100071 China

**Keywords:** Malaria, Children under 5, Female-headed household, Male-headed household, Nigeria

## Abstract

**Background:**

Nigeria is facing a severe malaria crisis, accounting for a significant proportion of global cases and deaths of malaria. This study aimed to investigate the differences between female-headed households (FHHs) and male-headed households (MHHs) and their impact on malaria risk among children under five (U5) in Nigeria.

**Methods:**

Data from the 2021 Nigeria Malaria Indicator Survey (NMIS) were used for this cross-sectional study. A representative sample of 10,988 households was analysed, with key variables subjected to frequency calculations, descriptive statistics, and bivariate analyses using t-tests and chi-square analyses to compare the differences between FHHs and MHHs.

**Results:**

Among all participants, 92.1% (N = 10,126) reported residing in male-headed households, while 7.8% (N = 862) reported living in female-headed households. MHHs were significantly more likely to own insecticide-treated bed nets (ITNs) than FHHs (64.7% vs. 53.6%, *P* < 0.001). U5 children in MHHs had a greater likelihood of sleeping under a bed net the night before the survey than U5 children in FHHs (35.3% vs. 30.0%, *P* < 0.05). The prevalence of fever in the previous two weeks among U5 children was similar in MHHs and FHHs (35.4% vs. 31.4%), and the testing rates for malaria among U5 children who experienced febrile episodes were higher in MHHs than FHHs (22.4% vs. 15.4%, *P* < 0.05). Although not statistically significant, FHHs exhibited a higher percentage of U5 children testing positive for malaria compared to MHHs (87.8% vs. 78.9%). On the other hand, FHHs had higher education levels, overall wealth index scores, and a larger presence in urban areas compared to MHHs (*P* < 0.001). Moreover, FHHs reported higher adherence to malaria prevention awareness (*P* < 0.001).

**Conclusion:**

In Nigeria, FHHs enjoy relatively better socioeconomic conditions and stronger awareness of malaria prevention compared to their male-headed counterparts. Contrary to expectations, FHHs are at an increased risk of malaria in children under 5 years old. This phenomenon is associated with entrenched gender inequality and the challenges women face in accessing critical assets. As women in FHHs bear the responsibility of income generation while caring for their children, it is crucial to prioritize interventions that address malaria management in FHHs to reduce both malaria incidence and mortality rates.

**Graphical Abstract:**

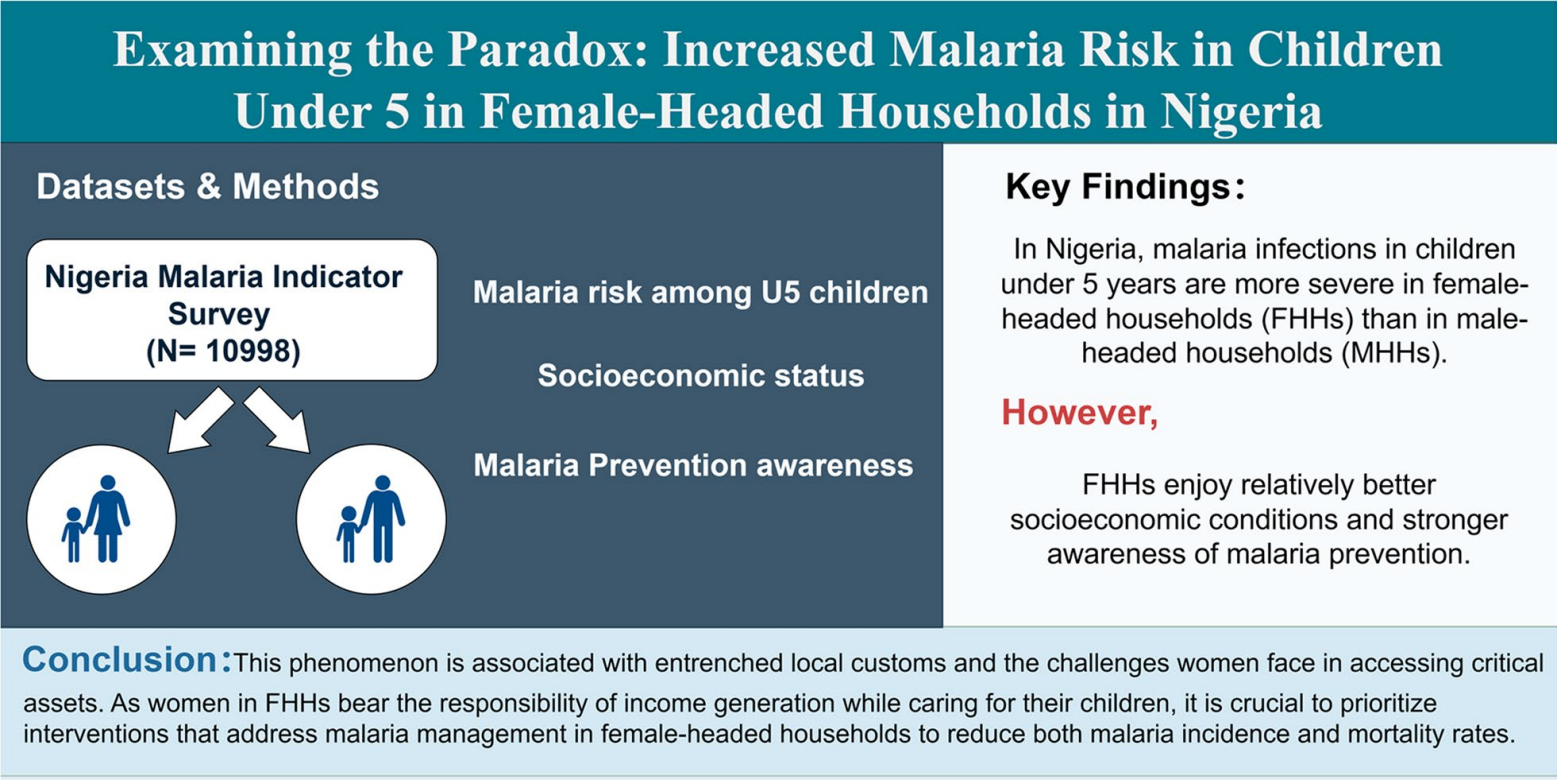

## Background

Malaria is a persistent and life-threatening disease that continues to pose a significant threat to global public health [1]. Sub-Saharan Africa is disproportionately affected, accounting for more than 90% of malaria cases and deaths [2]. Among the most vulnerable groups, children under 5 years old account for approximately 76% of all malaria-related deaths [3]. Nigeria, in particular, bears a substantial burden, with an estimated 27% of global malaria cases and 31% of global malaria deaths occurring within its borders [3].

The prevalence of malaria in any given area is influenced by the coexistence of vectors, transmission parasites, and susceptible human hosts, as well as the interactions between these three components [4, 5]. Recently, the role of environmental and demographic factors in malaria resurgence and local transmission has gained increased amounts of attention. Comprehensive analyses and studies on malaria control have revealed the significance of social, economic, and contextual variables [6–8]. Integrating data on social, cultural, economic, and environmental factors into risk models can enhance our understanding of malaria endemicity and inform long-term planning efforts [9, 10]. Furthermore, these studies deepen the understanding of the complex interactions involved in malaria incidence, prevalence, and transmission [11].

Despite increased support for malaria control programmes in Nigeria, the country continues to experience high prevalence and mortality rates [12–14]. This finding suggested that there are additional influential factors yet to be discovered. Identifying factors associated with perennial malaria transmission and prevalence in specific areas is crucial for targeting malaria hotspots and implementing effective control measures [10]. Unfortunately, only a limited number of studies have been conducted in Nigeria to address this issue [15, 16].

Poverty and gender are recognized as two factors associated with epidemic infectious diseases. The household-headship approach, which focuses on female-headed households as a measure of gender inequality and poverty, has been widely utilized in poverty studies [17]. Although female-headed households constitute a small proportion of all households, their share has been increasing globally over the past two decades [17]. However, research on the association between the sex distribution of household heads and malaria-related variables in children under five is scarce.

Therefore, this study aimed to investigate the impact of household head gender on malaria infection among children under 5 years old and the effectiveness of malaria prevention and testing in Nigeria. By filling this research gap, this study can gain valuable insights into the potential influence of household head gender on malaria outcomes and inform targeted interventions and policies for malaria control in Nigeria.

## Methods

### Data sources

The cross-sectional survey data utilized in this study were sourced from the 2021 Nigeria Malaria Indicator Survey (NMIS) conducted by the Demographic and Health Survey (DHS) program, which provides data on various health, population, and nutritional indicators across more than 90 countries [18]. The 2021 NMIS was the third survey of malaria indicators conducted in Nigeria, following those in 2010 and 2015. Its primary aim was to furnish current estimates of crucial demographic and health indicators related to malaria [19].

Specifically, the NMIS collected data on vector control interventions (such as mosquito nets), intermittent preventive treatment for malaria in pregnant women, exposure to malaria-related messages, care-seeking behaviour, treatment of fever in children, and social and behavioural change communication. Additionally, children aged 6–59 months underwent testing for anaemia and malaria infection [19]. The data collected through the NMIS are intended to aid policymakers and programme managers in the evaluation and design of initiatives and strategies aimed at enhancing the country’s population health. The NMIS is representative of a nationwide sampling of households, covering population and health issues pertinent to Nigeria [19].

### Study design

Children over 59 months of age were excluded from the study due to their exclusive focus on children under 5 years of age, including infants, who constitute the most vulnerable group, especially in areas with high transmission rates [20]. Additionally, the datasets were stratified to distinguish between household headships, specifically examining female-headed households (FHHs) and male-headed households (MHHs), with the aim of highlighting the disparities between these two types of household headships. Data analysis was conducted using Stata 18.0, employing analytical programs to compute frequencies and descriptive statistics for all key variables. Bivariate analyses comparing female-headed and male-headed households were performed using t-tests and chi-square analyses, with a *P* value of 0.05 considered the threshold for statistical significance.

### Socioeconomic status

In this study, socioeconomic status was comprehensively evaluated by educational attainment, household wealth and the urban living rate. Better socioeconomic status was defined as more urban residents living, greater educational attainment and higher wealth index scores. The educational level was classified into four groups (no education, primary, secondary, or higher), which were in accordance with the MIS definitions. MIS household wealth index scores are developed using principal component analysis, which typically includes variables describing durable asset ownership, access to utilities and infrastructure, and house construction materials [21]. The DHS and MIS classified the household wealth levels into five categories, namely, ‘‘poorest”, ‘‘poor”, ‘‘middle”, ‘‘rich”, and ‘‘richest”.

### Malaria risk in children under 5

MIS collects data on all of the internationally recognized malaria indicators [18]. Therefore, this study assessed malaria risk in children under 5 years old based on the following indicators: household ownership of insecticide-treated mosquito nets and their use, the proportion of children under 5 who slept under insecticide-treated nets the previous night, the fever rate among children under 5, the rate of malaria blood tests administered to children under 5 within 2 weeks prior to the survey, and diagnostic blood test results for children under 5 with fever.

### Malaria prevention awareness

The awareness of malaria prevention and control was also assessed via the Nigeria Malaria indicator survey (NMIS). In this study, the following variables were selected for comprehensive evaluation: take malaria prevention medication, sleep under ITN, use mosquito repellent, spray house with insecticide, fill in stagnant waters (puddles), keep surrounding clear, put mosquito screen on windows, other, and don’t know.

## Results

### FHHs are better off than MHHs

Among the identified individuals, 92.1% (N = 10,126) reported residing in male-headed households, while 7.8% (N = 862) reported living in female-headed households (Table 1). A comparison between female-headed households (FHHs) and male-headed households (MHHs) revealed that MHHs had a greater number of residents (*P* < 0.001) and a greater number of children under the age of 5 (*P* < 0.001). Conversely, compared with their male counterparts, FHHs were more frequently located in urban areas (*P* < 0.001), and female household heads generally exhibited greater educational attainment (χ^2^ = 329.9266, *df* = 3, *P* < 0.001). Moreover, FHHs demonstrated higher overall wealth index scores than MHHs did (χ^2^ = 164.5122, *df* = 4, *P* < 0.001) (Table 1).

**Table 1:** Household demographics from 2021 Nigeria Malaria Indicator Survey

Variable	Female-headed households (N = 862)	Male-headed households (N = 10126)	P value
Mean age (± SD)	42.5 (± 16.4)	42.9 (± 13.3)	< 0.001
Mean number of individuals in household (± SD)	5.7 (± 2.8)	7.6 (± 4.3)	< 0.001
Mean number of children under 59 months in household (± SD)	1.9 (± 1.0)	2.4 (± 1.4)	< 0.001
Rate in urban residence (%)	38.2 (329)	28.8 (2915)	< 0.001
Highest level of education (%)			< 0.001
No education	14.6 (126)	45.3 (4591)	
Primary	16.9 (146)	14.7 (1487)	
Secondary	51.5 (444)	30.0 (3035)	
Higher	16.9 (146)	10.0 (1013)	
Wealth index combined (%)			< 0.001
Poorest	8.1 (70)	21.4 (2167)	
Poorer	12.5 (108)	20.8 (2104)	
Middle	24.6 (212)	20.3 (2053)	
Richer	26.9 (232)	19.3 (1955)	
Richest	27.8 (240)	18.2 (1847)	

Data are presented as the mean ± SD or percentage (frequency). Mean age, Mean number of individuals in household, and Mean number of children under 59 months in household: t-tests; Rate in urban residence, Highest level of education, and Wealth index combined: chi-square tests

**Fig. 1:**
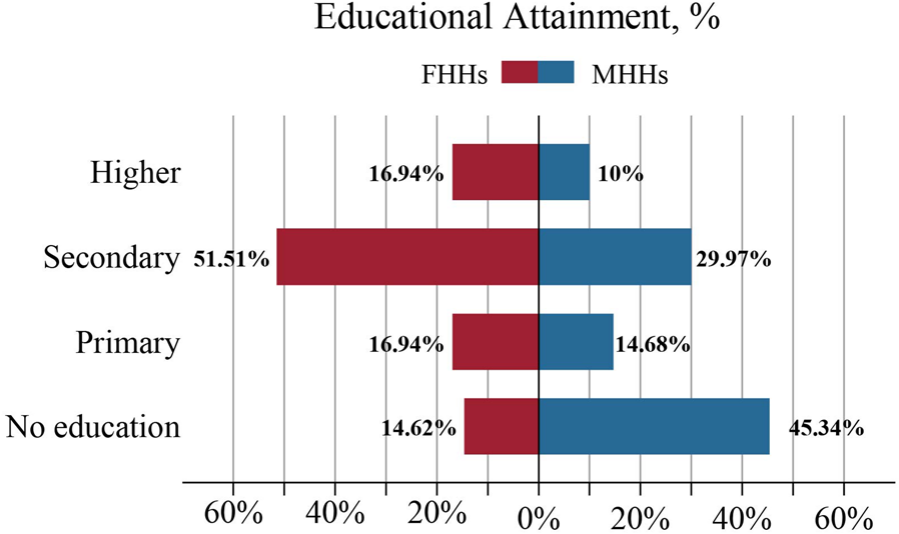
Differences in educational attainment between female-headed and male-headed households, 2021 Nigeria Malaria Indicator Survey. FHHs: female-headed households; MHHs: male-headed households

The distribution of individuals with no education, education below primary school or secondary or higher education was evaluated, and it is found that there were differences in education levels between female-headed households and male-headed households. Compared with women in female-headed households, women in male-headed households have lower education levels. Specifically, 45.3% of women in male-headed households lacked formal education, whereas 14.6% of women in female-headed households lacked formal education (Fig. 1).

**Fig. 2:**
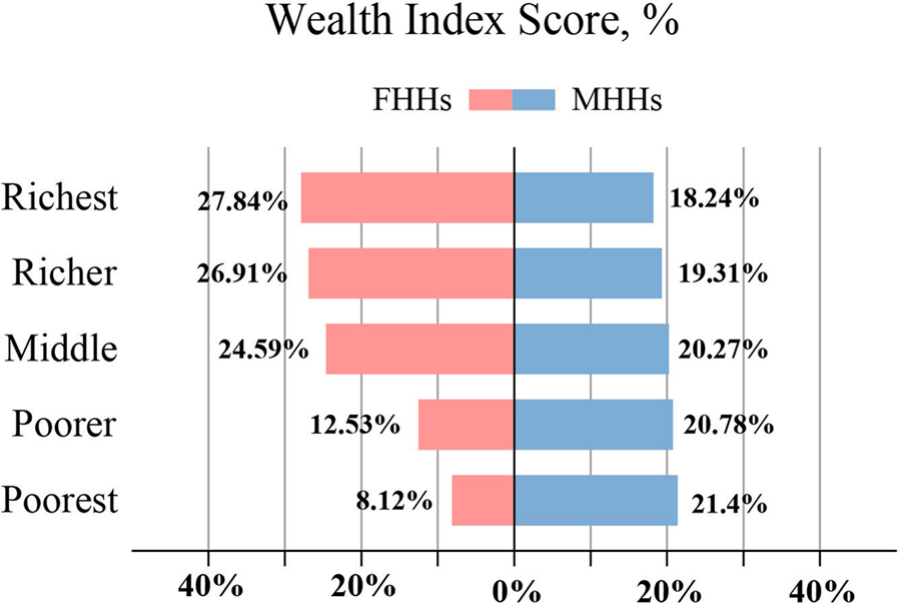
A comparison of wealth across female-headed and male-headed households, 2021 Nigeria Malaria Indicator Survey. FHHs: female-headed households; MHHs: male-headed households

The gap between female-headed households and male-headed households in terms of family wealth was judged by accumulating wealth index scores. The score is divided into five grades, namely, poorest, poorer, middle, rich and richest. Notably, compared with those of FHHs, the MHH families were more likely to be classified as "the poorest", with 21.4% of the male-headed families belonging to this category, while the proportion of female-headed families was only 8.12% (Fig. 2).

### Malaria infections in children under 5 years are more severe in FHHs than in MHHs

The assessment of the impact of the gender of the household head on malaria infections among children under 5 years old took into account the crucial malaria-related factors, such as the ownership of insecticide-treated nets, all children under 5 years old sleeping under such nets the previous night, and instances of fever in the past 2 weeks. The findings indicated that male-headed households are more likely to own insecticide-treated mosquito nets (64.7% vs 53.6%, χ^2^ = 42.62, *df* = 1, *P* < 0.001) and are more likely to report that all children under 5 years old slept under insecticide-treated mosquito nets the night before (35.3% vs 30.0%, χ^2^ = 68.9102, *df* = 3, *P* < 0.001) (Table 2).

**Table 2:** Malaria-related variables from the 2021 Nigeria Malaria Indicator Survey

Variable	Female-headed households (N = 862) (% (N))	Male-headed households (N = 10126) (% (N))	*P* value
Owns an ITN	53.6 (462)	64.7 (6554)	< 0.001
All children under 5 slept under an ITN last night	30.0 (249)	35.3 (3517)	< 0.001
Children under 5 with fever in the last 2 weeks before the survey	31.4 (266)	35.4 (3466)	0.062
Children under 5 with a fever who had a blood test for malaria	15.4 (41)	22.4 (776)	0.01
Children tested who tested positive for malaria	87.8 (36)	78.9 (612)	0.293

Data are presented as percentage (frequency). Bivariate analyses: chi-square tests. ITN indicates insecticide-treated net

In addition, the proportion of children under five years old who had a fever in the first two weeks was similar across female-headed and male-headed families (χ^2^ = 5.5708, *df* = 2, *P* = 0.062). In contrast, the proportion of individuals who sought malaria test was significantly greater among those with MHHs (15.4% vs 22.4%, χ^2^ = 9.1161, *df* = 2, *P* = 0.01). The percentage of female-headed households that tested positive for malaria was 8.9% greater than that of male-headed households, although the difference was not statistically significant (χ^2^ = 2.4573, *df* = 2, *P* = 0.293) (Table 2).

### FHHs may demonstrate more effective malaria prevention measures than MHHs

**Fig. 3:**
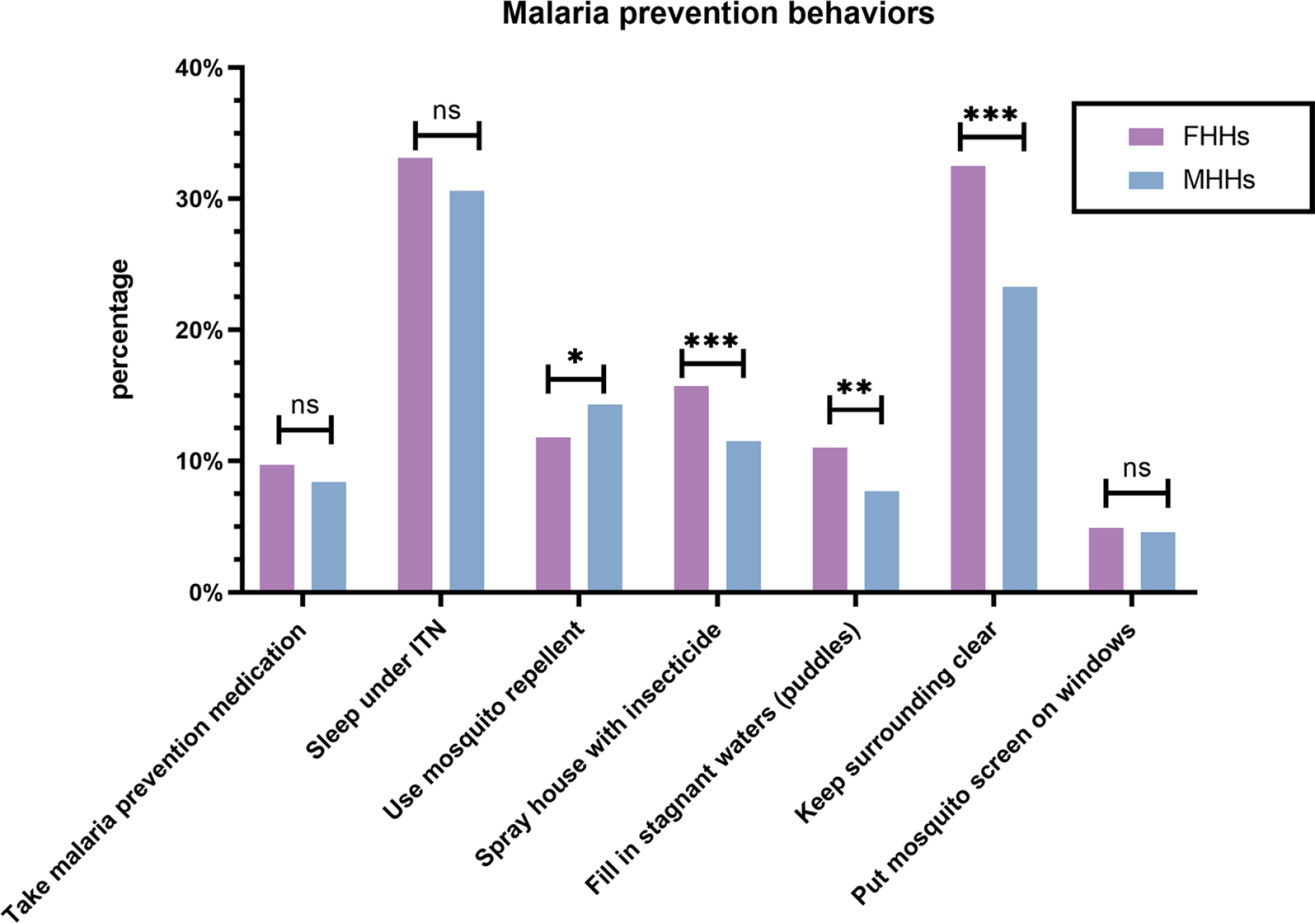
A comparison of malaria prevention awareness across household type, 2021 Nigeria Malaria Indicator Survey. FHHs: female-headed households; MHHs: male-headed households; ITN: insecticide-treated net (*P < 0.05, ** P < 0.01, *** P < 0.001, ns *P* > 0.05)

Next, the differences between male-headed and female-headed households in terms of their choice of preventive measures were analysed (Fig. 3). There was no statistically significant difference between female-headed households (FHHs) and male-headed households (MHHs) in terms of the adoption of three preventive measures: taking malaria prevention medication (χ^2^ = 0.892, *df* = 1, *P* = 0.169), putting mosquito screens on windows (χ^2^ = 0.0953, *df* = 2, *P* = 0.758), and sleeping under the ITNs (χ^2^ = 2.3646, df = 1, *P* = 0.124) (Table 3).

**Table 3:** Malaria prevention behaviors by household type, 2021 Nigeria Malaria Indicator Survey

Malaria prevention variable	Female-headed households (N = 862) % (N)	Male-headed households (N = 10126) % (N)	*P* value
Take malaria prevention medication	9.7 (84)	8.4 (849)	0.169
Sleep under ITN	33.1 (285)	30.6 (3093)	0.124
Used mosquito repellent	11.8 (102)	14.3 (1447)	0.047
Spray house with insecticide	15.7 (135)	11.5 (1163)	< 0.001
Fill in stagnant waters (puddles)	11.0 (95)	7.7 (778)	0.001
Keep surrounding clear	32.5 (280)	23.3 (2354)	< 0.001
Put mosquito screen on windows	4.9 (42)	4.6 (470)	0.758
Other	3.9 (34)	2.4 (246)	0.007
Don’t know	10.3 (89)	6.4 (645)	< 0.001

Data are presented as percentage (frequency). Bivariate analyses: chi-square tests. ITN indicates insecticide-treated net

In comparison to male-headed households (MHHs), female-headed households (FHHs) demonstrate a preference for spraying houses with insecticides, filling in stagnant waters (puddles), and maintaining clear surroundings, with statistical significance indicated by *P* < 0.001. Additionally, relative to FHHs, MHHs display a greater preference for using mosquito repellents, and a greater proportion lack awareness of malaria prevention methods (Table 3).

## Discussion

This study aimed to compare male-headed households (MHHs) and female-headed households (FHHs) in Nigeria regarding their malaria prevention practices and infection rates. The findings reveal some intriguing conclusions that shed light on the complex dynamics between household types and malaria risk.

The analysis showed that MHHs had greater ownership of insecticide-treated nets (ITNs) and a greater number of children sleeping under ITNs, indicating that MHHs performed better than FHHs in terms of malaria prevention. These findings align with previous studies highlighting the importance of ITNs in reducing malaria transmission [22, 23]. The higher rates of testing for malaria among febrile children in MHHs further support the notion that these households are more proactive in seeking appropriate healthcare when needed. Moreover, the higher rate of positive malaria tests in MHHs suggests that children under the age of five in FHHs are at greater risk of malaria infection. These results emphasize the vulnerability of FHHs and the need for targeted interventions to address this disparity.

Contrary to the initial expectations, FHHs exhibited higher educational attainment, wealth index scores, and urban residence. These factors are commonly associated with better socioeconomic status and improved access to healthcare services [24, 25]. However, this approach does not translate into superior malaria prevention and testing capabilities within FHHs. It is possible that additional considerations, such as access to healthcare facilities, quality of care, and cultural norms, contribute to the observed disparities between household types.

To assess poverty status across household types, MIS employed the consumption expenditure approach [21]. The consumption expenditure approach primarily focuses on economic indicators, such as income and consumption. In contrast, an alternative approach, the livelihoods approach, involves examining the multidimensional nature of living conditions, encompassing aspects such as access to assets, social networks, and capabilities (such as education, skills, and health) [26]. Through this framework, Kpoor et al*.* discovered that FHHs lack access to essential assets, indicating that they are not as economically secure as MHHs [27]. The findings in this study confirmed that FHHs lack access to essential assets and are economically less secure than MHHs are. These results highlight the importance of adopting a multidimensional perspective when evaluating poverty and its implications for health outcomes.

The gender dynamics within households play a significant role in decision-making processes and resource allocation [28, 29]. Sub-Saharan Africa (SSA) stands out as one of the regions with the highest levels of gender inequality [30]. Women face systematic disadvantages in accessing education, owning assets, and pursuing economic opportunities [31–33]. Furthermore, in Nigeria, traditional norms typically designate men as household heads, with some exceptions [12]. This study underscores the need to recognize the diverse nature of FHHs, ranging from young mothers to older women with grown children [34]. The assets and resources available to these households can vary significantly, further complicating the relationship between household type and malaria risk.

One intriguing finding is that FHHs exhibited better awareness of preventive measures, such as sleeping under ITNs and engaging in environmental sanitation. However, these behaviours did not correlate with reduced malaria infection rates. This discrepancy suggests that factors beyond individual-level practices, such as vector control strategies, healthcare access, and community-level interventions, may influence malaria transmission dynamics in these households [35, 36].

Moving forward, the implementation of culturally appropriate, sustainable, and effective interventions is crucial for successful malaria control strategies. Mosquirix, the first malaria vaccine recommended by WHO, also known as RTS,S/AS01, has successfully reduced early childhood mortality in Ghana, Kenya and Malawi by 13%, but it has only modest efficacy and its protection soon wanes [3]. Now, a second vaccine is poised to join the fight, with the approval by the World Health Organization (WHO) of a shot called R21/MatrixM [37]. Similar to RTS,S/AS01 in design, it can be produced more cheaply and in greater quantities [38]. It should help fill the huge gap between supply and demand for malaria vaccines, potentially preventing tens of thousands of children’s deaths a year. The R21/MatrixM vaccine was highlighted as a 2023 Breakthrough Of The Year by Science Journal, being lauded as ‘‘New hope against malaria.’’ [39]. If the vaccine is widely used and combined with other recommended malaria control interventions, it is expected to have a significant impact on public health [37]. Widespread adoption and uptake among both MHHs and FHHs are necessary to effectively reduce malaria-related mortality in children under five years of age.

Several limitations should be acknowledged. First, relying on self-reported data introduces potential sources of bias, including recall bias and social desirability bias. Additionally, the seasonal nature of malaria and the timing of data collection may have impacted the outcomes. Moreover, the limited number of households with febrile children seeking malaria testing might have affected the statistical power, warranting cautious interpretation of the results.

In summary, this study provides valuable insights into the differences between MHHs and FHHs regarding malaria prevention practices and infection rates. This highlights the vulnerability of children under five in FHHs and the need for targeted interventions to address this disparity. The findings underscore the importance of considering multiple dimensions of poverty and the complex interplay between household type, socioeconomic factors, and cultural norms. Moving forward, a comprehensive approach that integrates preventive measures, healthcare access, and community-level interventions is essential for effective malaria control strategies in Nigeria.

## Conclusion

In Nigeria, female-headed households enjoy relatively better socioeconomic conditions than do their male-headed counterparts. Contrary to expectations, FHHs are at increased risk of malaria in children under 5 years old. This suggests that the challenges related to malaria prevention, contraction, and management are widespread and particularly demanding for FHHs in Nigeria. This phenomenon is associated with entrenched local customs and the challenges women face in accessing critical resources. As women in FHHs bear the responsibility of income generation while caring for their children, it is crucial to prioritize interventions that address malaria management in female-headed households to reduce both malaria incidence and mortality rates.

## Data Availability

The data that support the findings of this study are available from the 2021 Nigeria Malaria Indicator Survey (NMIS), which was conducted by the Demographic and Health Survey (DHS) program. But restrictions apply to the availability of these data, which were used under license for the current study, and so are not publicly available. Data are however available from the authors upon reasonable request and with permission of the Demographic and Health Survey (DHS) program.

## Abbreviations

FHHs: Female-headed households
MHHs: Male-headed households
U5: Children under 5
NMIS: 2021 Nigeria Malaria Indicator Survey
DHS: Demographic and Health Survey
ITNs: Insecticide-treated nets
